# A retrosynthetic biology approach to metabolic pathway design for therapeutic production

**DOI:** 10.1186/1752-0509-5-122

**Published:** 2011-08-05

**Authors:** Pablo Carbonell, Anne-Gaëlle Planson, Davide Fichera, Jean-Loup Faulon

**Affiliations:** 1 iSSB, Institute of Systems and Synthetic Biology, University of Evry, Genopole Campus 1, Genavenir 6, 5 rue Henri Desbruères, 91030 EVRY Cedex, France

## Abstract

**Background:**

Synthetic biology is used to develop cell factories for production of chemicals by constructively importing heterologous pathways into industrial microorganisms. In this work we present a retrosynthetic approach to the production of therapeutics with the goal of developing an *in situ *drug delivery device in host cells. Retrosynthesis, a concept originally proposed for synthetic chemistry, iteratively applies reversed chemical transformations (reversed enzyme-catalyzed reactions in the metabolic space) starting from a target product to reach precursors that are endogenous to the chassis. So far, a wider adoption of retrosynthesis into the manufacturing pipeline has been hindered by the complexity of enumerating all feasible biosynthetic pathways for a given compound.

**Results:**

In our method, we efficiently address the complexity problem by coding substrates, products and reactions into molecular signatures. Metabolic maps are represented using hypergraphs and the complexity is controlled by varying the specificity of the molecular signature. Furthermore, our method enables candidate pathways to be ranked to determine which ones are best to engineer. The proposed ranking function can integrate data from different sources such as host compatibility for inserted genes, the estimation of steady-state fluxes from the genome-wide reconstruction of the organism's metabolism, or the estimation of metabolite toxicity from experimental assays. We use several machine-learning tools in order to estimate enzyme activity and reaction efficiency at each step of the identified pathways. Examples of production in bacteria and yeast for two antibiotics and for one antitumor agent, as well as for several essential metabolites are outlined.

**Conclusions:**

We present here a unified framework that integrates diverse techniques involved in the design of heterologous biosynthetic pathways through a retrosynthetic approach in the reaction signature space. Our engineering methodology enables the flexible design of industrial microorganisms for the efficient on-demand production of chemical compounds with therapeutic applications.

## Background

Synthetic biology is being used for therapeutic production either to develop cell factories using industrial microorganisms [1,2] or to synthesize genetic circuits allowing *in situ *therapeutic delivery [3]. Recombinant DNA technology has already provided the ability to genetically engineer cell strains in order to import pathways from other organisms capable of producing small molecule chemicals into microbial chassis. Moreover, to estimate the efficiency of the overall process, metabolic engineering-based tools consider models of cell metabolism as a whole, allowing the identification and redesign of bottlenecks in the biosynthetic pathways. Therefore, the next challenge ahead remains the integration of all these design steps into a flexible and automated biosynthetic manufacturing pipeline of molecules.

In recent years, many successful examples of bioproduction of chemicals with therapeutic interest through metabolic engineering have been reported. Among others, plant secondary metabolites that are of medicinal value, such as the terpenoids artemisinic acid [4] and paclitaxel (taxol) [5], benzylisoquinoline alkaloids [6], and flavonoids [7,8] have been successfully produced by metabolically engineered microorganisms. Similarly, heterologous production of therapeutically important antibiotics such as aminoglycosides derivatives, which include ribostamycin [9], neomycin, gentamicin and kanamycin, as well as other natural products like polyketides [10,11] and nonribosomal peptides [12] have been reported. Flexible production of novel antibiotics is of special interest in order to fight against the increasing emergence of multidrug-resistant pathogens [13-15].

In an attempt to rationalize the biosynthetic design process, metabolic engineering models the metabolic network of the cell as a whole [16,17]. A suitable topological representation of the metabolic network can be achieved by using directed hypergraphs [18,19] where catalytic reactions are hyperedges connecting node substrates to products. Moreover, genome-wide reconstructions of an organism's metabolism with explicit reference to the stoichiometry of the reactions can be studied in order to estimate steady-state fluxes [20]. Sensitivity analysis of fluxes provides a systematic way to determine production bottlenecks, where gene overexpression or repression might enhance production for the target compound [21,22]. In addition, stochastic and deterministic system dynamics methods are used to simulate enzymatic reaction kinetics [23].

Through metabolic modeling, the repertoire of biochemical transformations in *de novo *biosynthetic pathways are extended beyond what is present in metabolic databases like KEGG [24] and MetaCyc [25]. *In silico *methods for *de novo *pathway prediction and optimization are mainly based on two approaches: homologies of chemical structure transformation patterns [26-29], and knowledge-based expert systems [30,31]. Retrosynthesis algorithms [32] perform a backward search for biosynthetic routes leading from a target compound to the host metabolites through iterative application of a defined set of biochemical transformation rules. One approach is BNICE [33], where molecules and reactions are represented by bond-electron matrices (BEM) [34]. BEM entries correspond to the covalent bond order between atoms, whereas the Dugundji-Ugi model for a metabolic reaction is implemented through the matrix difference between the BEM of products and substrates. With BNICE, reactions in the KEGG database [24] are represented through approximately 250 unique elementary transformations, which approximately correspond to the classification at the 3rd EC digits [35,36]. In the same fashion, the molecular signature descriptor [37] is an algorithm that returns for a given target compound fourth and third EC digits, respectively, of predicted enzymes capable of producing the structure. Similarly, other retrosynthetic framework has been proposed based on 50 reaction rules [38].

A retrosynthetic search in the metabolic hypergraph might lead to a combinatorial explosion. For instance, using only 50 reaction rules, 100,000 reaction routes were predicted for the production of isobutanol [38], far more than what could be realistically tested in the laboratory. Thus, in order to find a trade-off between the inherent complexity of *de novo *pathway design and the use of experimental information, we present here a tool based on the coding of compounds and reactions through molecular signatures [39]. The molecular signature is a canonical representation of the subgraph surrounding a particular atom in a molecular structure up to a predefined diameter or height *h*. A metabolic reaction signature is given by the difference between the signatures of products and substrates [40]. As further described in the Methods section, the signature coding system can be made more or less specific to compounds and reactions by selecting the height, low heights are less specific (as molecular signatures become more and more ambiguous) and high heights are specific (as molecular signatures become more and more precise), thus the numbers of *de novo *reactions and consequently *de novo *pathways can be controlled.

Once metabolic models for the heterologous biosynthesis of target compounds have been determined, individual performances for the predicted pathways need to be characterized in order to prioritize the engineering of the most promising routes into the chassis organism. Several computational frameworks have proposed different factors that might influence the performance of an engineered strain. PathMiner introduced a path cost associated with the number of heterologous enzymes measured through a chemical distance [41]. BNICE applied the group contribution method [42] for reactants and products in order to rank pathways based on the thermodynamic favorability [43]. Other aspects influencing the pathway performance are pathway length, organism specificity [38], heterologous expression, growth rate, and precursor supply [44]. In addition, many other factors might be considered, for instance, PathoLogic defined 123 pathway features that may be relevant to the pathway ranking problem [31]. Therefore, subsequent optimization of the heterologous engineered strain through genetic, metabolic and enzyme design approaches would be usually necessary in order to attain the desired final yields in the production of the target compound. Moreover, increasing efficiency levels for rate-limiting enzymatic reactions involved in the pathway is an additional strategy for the rational design of industrial strains [37,45,46]. We present here a unified framework that combines several techniques involved in the design of heterologous biosynthetic pathways through a retrosynthetic approach in the reaction signature space, enabling the flexible design of industrial microorganisms for the efficient on-demand production of chemical compounds of interest.

## Results and Discussion

### The extended metabolic reaction space (EMRS)

**Figure 1:**
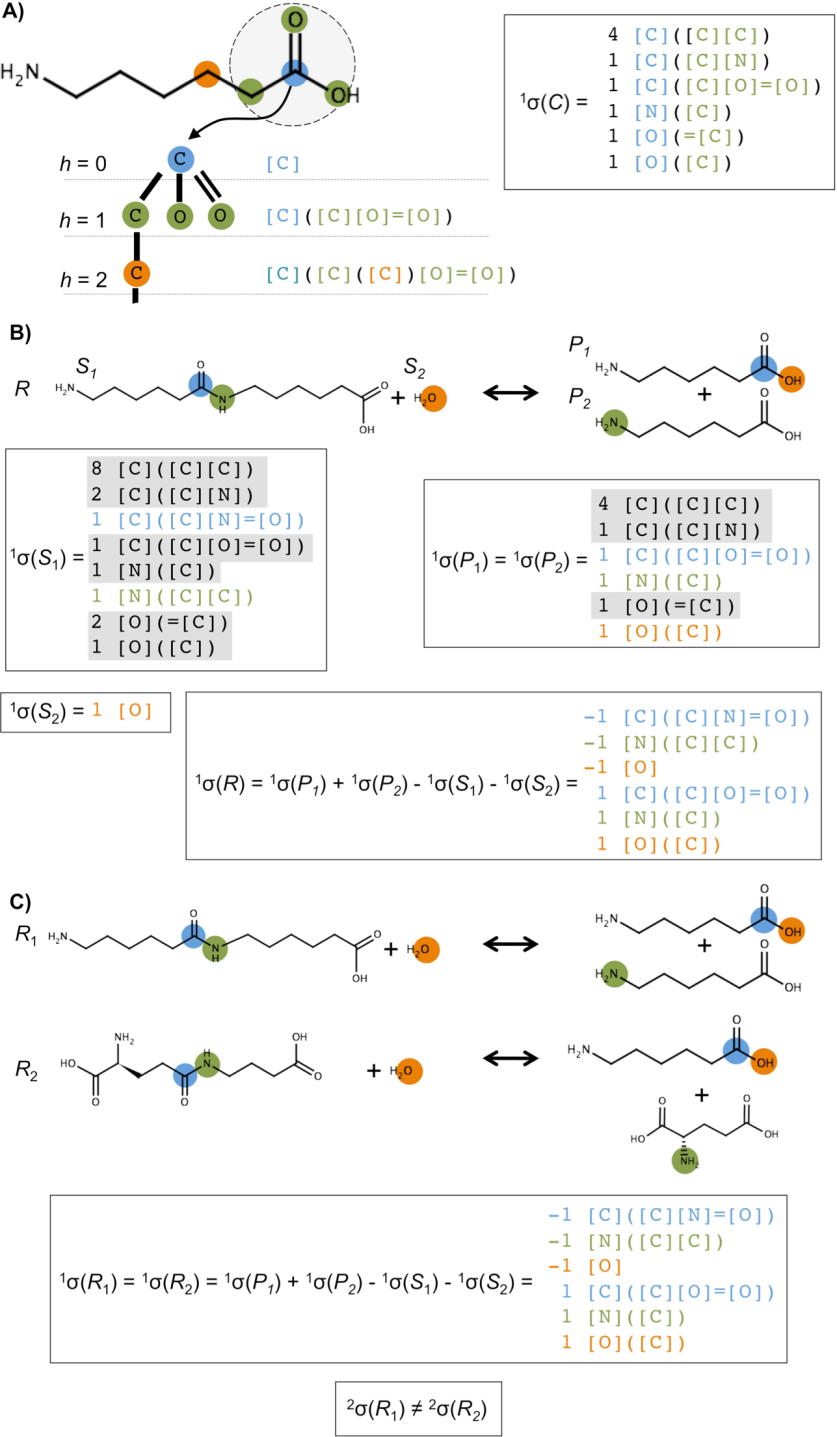
**The atomic, molecular and reaction signature coding**. A) The process for computing the molecular signature for a compound *C *is illustrated for 6-aminohexanate. The process starts by computing the atomic signature for each atom. In the given example, the atomic signature for the carbon in the carboxylic group is computed up to height *h *= 2. At height *h *= 0 (blue), the molecular graph rooted at the atom is given by the atom itself; at height *h *= 1 (green) a canonical representation of the root atom and its first atomic neighbors are given; the process continues similarly for heights *h *= 2 (orange) and higher until the diameter of the graph is reached. Atomic signatures are collected for all atoms and sorted in order to provide the molecular signature, for instance the molecular signature ^1^*σ*(*C*) of height *h *= 1 is given at the left; B) The coding of reactions signatures is illustrated for the 6-aminohexanoate hydrolase (EC 3.5.1.46). The reaction signature contains the net difference between the products and the substrates. In the figure, the reaction signature ^1^*σ*(*R*) was computed for height *h *= 1; C) Illustration of how signatures of reactions provide a way to measure their chemical similarity. For example, the previous reaction (EC 3.5.1.46) has the same signature at height *h *= 1 than 4-(*γ*-glutamylamino)butanoate amidohydrolase (EC 3.5.1.94). However, both signatures differ at height *h *= 2, having in this case a Tanimoto similarity of ^2^*s*(*R*_1_, *R*_2_) = 0.81 (see Equation 14 in Methods).

**Figure 2:**
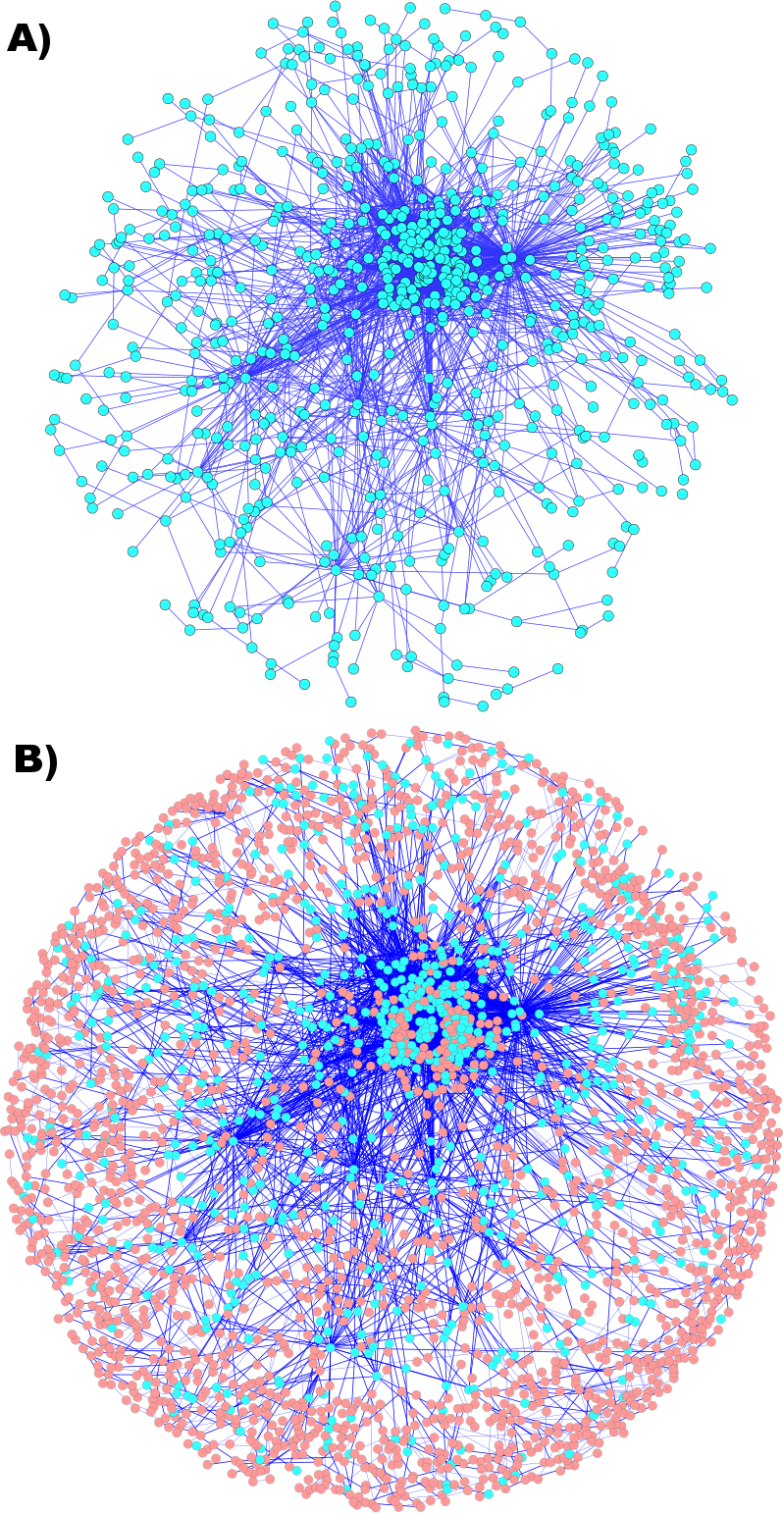
**Metabolic networks in the EMRS**. A) Metabolic reaction map of *E. coli*, where endogenous metabolites are depicted as light blue nodes connected by edges representing reactions; B) Retrosynthetic map containing reachable compounds in the *E. coli *EMRS through exogenous reactions, where exogenous metabolites are represented by pink nodes connected through reactions (thin edges) to the *E. coli *network. There are 966 endogenous and 2338 exogenous compounds, respectively, that can be reached through reactions in the EMRS. There are 4,344 edges connecting endogenous compounds and 8,931 edges leading to exogenous compounds.

Our method starts by mapping the metabolic network into the signature space in order to build an extended representation of the metabolic reaction space (see definitions in Methods). Molecular signatures, which are a representation of the molecular graph, can be used in order to code reactions [46]. This process is illustrated in Figure 1. Canonical molecular signatures identify unique compounds when they are computed at the height *h *of the maximum diameter of the molecular graph, whereas signatures at lower height *h *provide a way to search for and enumerate similar chemical compounds. Likewise, reaction signatures, which are biochemical reactions coded into the molecular signature representation (Equation 7 in Methods), are used in order to search for and enumerate similar reactions. We define the extended metabolic reaction space (EMRS) as the set of all possible reactions that can be generated from signatures contained in the metabolic network. Therefore, the EMRS consists of both reactions in the metabolic network and additional putative reactions, which are assumed to be promiscuously catalyzed by enzymes present in the organism. Given a finite height *h*, novel reactions are discovered through this method by performing a search in the metabolite signature space of combinations of stoichiometric coefficients of metabolites having the same signature as either the substrates or the products. Figure 2 shows the metabolic reaction map of the 966 endogenous metabolites in *E. coli *(Figure 2A) and of the additional 2338 compounds that are reachable from *E. coli *after the generation of the EMRS (Figure 2B).

An illustrative example is the metabolite signature space of height *h *= 0. In this space, compounds are represented by their elemental formula. Similarly, the combination of the substrates and products in the reaction are represented by their total molecular formula. Thus, any combination of compounds satisfying the elemental formula is considered a putative set of reactants. In order to compute the EMRS of height *h *= 0, we need to solve Diophantine equations in the signature space of height *h *= 0 that generally lead to a set of solutions too large to be of use. In the case of *h *= 1, the number of newly created reactions is still significantly large (for a network like the one shown in Figure 2, it would be above 10^6^). This number, nevertheless, becomes tractable once we consider heights higher than *h *= 2, which corresponds to a 17.72% increase in the number of reactions with respect to nominal reactions, as it is shown in Table 1. Starting from the list of coded reactions, the iterative backward application of the biochemical transformations to compounds of interest allows the identification of enzymatic routes linking the desired compound to precursors that are endogenous to the chassis organism. Each of these routes constitutes an exogenous biosynthetic pathway for that compound. In the next sections, we present an approach for ranking the biosynthetic pathways of a given compound in order to select the best pathways to engineer in the chassis organism.

**Table 1:** Reactions in the EMRS

height *h*	reactions	% increase from canonical
2	9083	17.72%

3	7882	2.15%

4	7800	1.09%

5	7752	0.47%

6	7725	0.12%

canonical	7716	0%

Number of novel generated putative reactions in the EMRS for different heights *h*.

### Decision flowchart for selecting and ranking best pathways

**Figure 3:**
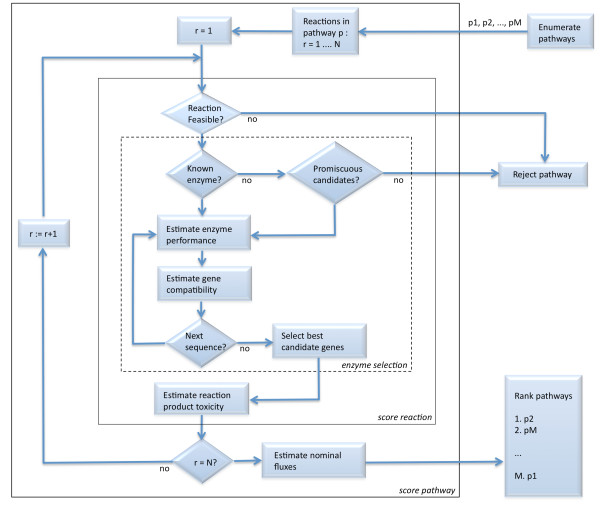
**Flowchart for ranking pathways**. In order to rank pathways, each reaction *r *= 1 ... *N *in the enumerated pathways *p*_1 _... *p_M _*is first tested for thermodynamical feasibility; enzyme candidates are subsequently tested for performance and homogeneity so that the one with the lowest cost is selected; the cost of toxicity of each reaction product is then added; finally, the nominal flux is estimated for the overall pathway.

The EMRS introduces putative novel reactions that share the same signature as their parent nominal reactions at the chosen height *h *of molecular resolution (Equation 8 in Methods). Those putative reactions generated by our molecular signature-based algorithm need to be validated and ranked. Figure 3 shows the decision flowchart used in our approach in order to accept or reject putative reactions in a pathway as well as to score its overall performance once inserted into the chassis organism. Reactions are first tested for their thermodynamic feasibility. Next, if no known enzyme sequences are available in the database, the enzyme sequence space is searched in order to find candidate sequences that might catalyze the given reaction as a promiscuous activity. Gene compatibility, enzymatic performance, toxicity of products and steady state fluxes are finally estimated in order to score the pathway.

We introduce the following function to quantify the cost of inserting an exogenous enzyme sequence *S_i _*processing the reaction r* in the pathway:(1)

where promis(*S_i_*) is the predicted enzyme promiscuity for the sequence *S_i_*, perf(r*, *S_i_*) is the estimated catalytic performance of the given sequence *S_i _*for reaction r*, and het(*S_i_*) is the gene compatibility of the sequence *S_i_*. *ω_p_*, *ω_e _*are parameters used in order to weight the contribution of each term to the cost function. All three terms are normalized before entering the expression so that each score is always given by a value in the same range between 0 and 1. Therefore, the cost function in Equation 1 is always defined positive and bounded. Promiscuity contributes negatively to the cost function because enzymes with higher level of promiscuity are considered better candidates for catalyzing the desired transformation r* as a side reaction. In the same fashion, enzyme performance contributes negatively since reactions with higher performance are considered less expensive in terms of the cost of insertion. Once all terms are defined for each reaction r* in the EMRS, this strategy enables the determination of those gene sequences S* that minimize the insertion cost:(2)

Furthermore, several additional adverse effects may be hindering the successful expression and performance of inserted enzymes, while the toxicity of intermediate metabolites might impede cell survival and growth. These effects need to be taken into account in order to rank the pathways. For instance, an estimate of toxicity values (IC50 or half minimal inhibitory concentration) for the intermediates *p *in the chassis organism, may be obtained either from experimental databases [47] or from structure-activity relationship models [48]. In addition, compound yields from inserted pathways are rarely additive, since other routes may be competing with the target pathway and inhibiting the production of the desired compound [5]. Here we use a multi-criteria approach in order to score the cost of pathway insertion with respect to the general goal of producing a target molecule *c*, with a cost function defined as follows:(3)

where *W*(*c*, *ρ*) considers the following effects: *v_c_*(*ρ*), nominal yield of compound *c *in pathway *ρ*; , minimum cost of insertion of each enzyme in the pathway as given by Equation 1; and tox(*p*), the inverse of the IC50 value. Parameters (*λ*_flux_, *λ*_path_, *λ*_tox_) need to be adjusted in function of the desired weight given to the costs of pathway insertion and metabolite toxicity. In our method, these parameters were optimized so that pathways that are fully annotated in the reference database, for instance KEGG, are ranked first with respect to predicted pathways (see details in Methods).

The minimum of this cost function *W*(*c*, ρ*(*c*)) at the optimal pathway:(4)

provides a trade-off between the simultaneous goals of obtaining the maximum nominal yield while keeping the overall process efficient and side effects attenuated. In the following sections, we present our approach in order to quantify each term in the pathway cost function *W*(*c*, *ρ*) in Equation 3.

### Predicting enzyme activity in promiscuous putative reactions

Our method provides for each reaction in the EMRS a ranked list of candidate sequences, as given by the score in Equation 1, along with their predicted catalytic efficiencies. When there is no sequence information in databases about enzymes catalyzing the desired reaction r*, we must rely on the prediction of enzymes as putative candidates to process other substrates (multispecificity) or to catalyze a promiscuous reaction other than their native ones [49]. Furthermore the thermodynamic feasibility of that reactions as well as the performance of the predicted promiscuous enzymes need to be evaluated. These preliminary evaluations, which are described next, are carried out in order to implement an early rejection of false hits, as shown in the flowchart of Figure 3.

#### Thermodynamic feasibility

Putative reactions need to be validated for their directionality or thermodynamic feasibility. We performed this validation assuming that the metabolites' concentration are spatially invariant and that temperature and pressure are constant. Under these assumptions, standard Gibbs free energies of reactions can be estimated using a group contribution approach [43,50]. Only reactions estimated to be thermodynamically feasible are added to the EMRS.

#### Enzyme promiscuity

As part of our methodology for biosynthetic pathway design, candidate enzyme sequences catalyzing feasible reactions in the EMRS have to be identified from the set of known enzymes. Namely, our procedure for extending the metabolic network relied on the underlying assumption that reactions with the same signature are likely to be processed by similar enzyme sequences [37], which in turn implies that the ability to catalyze the putative reaction is already present in the enzyme in the form of latent promiscuous activity. We have shown in a previous study [46] that enzyme multispecificity and promiscuity are properties that can be characterized by using a molecular signature representation. Thus, those multiple reactions generated in the EMRS from a nominal parent reaction can be interpreted as promiscuous activities predicted to be present in the set of known enzymes. Therefore, as shown in the decision flowchart in Figure 3, in order to consider a given sequence as a potential candidate that processes the putative reactions, a preliminary requirement has been introduced in the decision chart so that the enzyme should exhibit promiscuous activity based on the estimations performed by a molecular signature-based predictor (see Methods).

#### Tensor product

The next step consists of searching for candidate enzymes to process a given reaction in the EMRS. In case that no known enzyme sequences were available for the reaction, candidate enzymes were determined by a signature-based enzyme-reaction predictor by following the procedure known as the tensor product [51]. We assumed that best candidate enzyme sequences for a putative reaction were more likely to belong to the list of sequences known to catalyze reactions that are chemically similar to the given reaction.

Therefore, reactions generated by the enumeration algorithm in the EMRS were first clustered into groups of similar reactions by a distance metrics, which was defined as the Tanimoto similarity of reaction signatures [52]. The tensor product procedure (see Methods) was then used in order to locate best enzyme sequence candidates within the reaction cluster.

#### Enzyme performance

In addition, performance of exogenous enzymes needs to be evaluated. We have developed a decision tree learning method to estimate enzyme activity [53] using kinetics information from the BRENDA database [54] (turnover rates, Michaelis constant *K_M _*, and inhibition constant *K_i_*). As shown in Figure 3, predictions of enzyme performance perf(r*, *S_i_*) for the list of candidate enzymes entered into our decision flowchart in order to score the sequences in Equation 1.

### Quantifying the compatibility between the host and heterologous genes

Another aspect to be addressed when considering the overall enzyme cost defined in Equation 1, is the effect of inserting heterologous genes, since the diversity of base-pair content is organism-specific. By minimizing this difference, expression levels can be maximized [55]. In order to quantify the compatibility between the host and heterologous genes, we have implemented a machine-learning approach based on several descriptors: gene sequence descriptors (sequence length, GC content); organism specificity (phylogenetic distance between source organism and chassis); probability of protein expression as inclusion bodies; protein descriptors (percentage of hydrophobic and charged amino acids); and secondary structure distribution. These descriptors were computed for the entire KEGG database of non-redundant enzyme sequences and then used in order to train support vector machine-based predictors for the chassis organisms of interest (see Methods).

Furthermore, the successful expression of a heterologous gene depends on several additional sequence-independent factors, such as an adequate selection of promoters, RBS, and codons [56]. In some cases, we should also consider the need for some other type of specific modifications depending strictly on the type of compound to be synthesized, such as protein engineering of P450s [57] or modular design for the complex assembly machinery involved in the production of secondary metabolites like polyketides [58] and nonribosomal peptides [59,60], which need to be evaluated on a case-by-case basis in order to rank and select the best genes to engineer.

### Predicting compound toxicity

Exogenous enzymes inserted in the chassis might catalyze reactions synthesizing new products in the organism. As a side effect, however, intermediate metabolites involved in the exogenous pathways as well as any other side product of the new reactions may induce undesired toxic responses in the cell. Therefore, it is necessary to consider toxicity effects of the compounds. For this purpose, we have developed a structure-activity relationship model based on a library of 150 tested compounds covering a wide range of toxicity levels [61]. The model was built by using several molecular descriptors including molecular signatures as input descriptors, achieving a performance of *Q*^2 ^= 0.68. For any given reaction in the EMRS, toxicity was given by the sum of the predicted toxicity for each product, allowing us to identify pathways involving highly toxic metabolites in order to rank them with lower score.

Special consideration when predicting compound toxicity should be given to those cases when genes encoding for resistance to the compound are going to be engineered as part of the biosynthetic gene cluster. For instance, when producing an antibiotic in *E. coli *such as penicillin, it is necessary to introduce genes that code for *β*-lactam resistance in the organism in order to make bacteria immune to that antibiotic. Therefore, if resistance to some product is going to be inserted into the strain, the attenuation effects in toxicity for that family of compounds has to be updated into the model.

### Estimation of nominal fluxes

The insertion of new reactions into the chassis organism can perturb its metabolic network and therefore the equilibrium of the steady-state fluxes might be altered [20]. By using a constraints-based flux analysis on a genome-wide reconstructed metabolic model of the engineered strain, we obtain the solution space within cell's capacity. Our objective is to maximize the production of the desired compound while keeping cell growth. For each engineered strain, we obtained an estimate of expected net yield of product, which is not further metabolized, at the given controlled media conditions. In addition, flux balance analysis is a flexible analytical technique that can also be applied in other ways in our design framework for biosynthetic pathways. For instance, it can be used in a systematic way in order to perform a sensitivity analysis to determine production bottlenecks, where overexpression and gene knockouts might enhance production for the target compound [21,22].

### Pathway enumeration and optimal search in the EMRS

**Figure 4:**
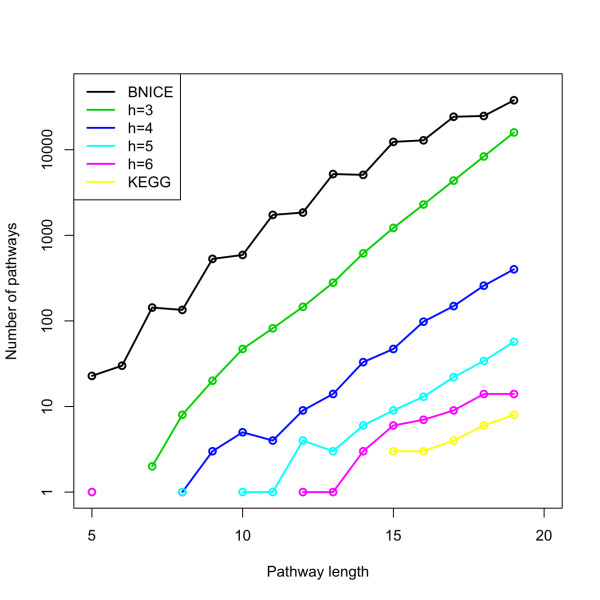
**Controlling the complexity of the pathway enumeration problem through molecular signatures**. Comparison between pathway length distributions between tyrosine and chorismate for novel reactions generated by the BEM representation (BNICE) [33], the EMRS of heights *h *= 3 to 6, and the original reactions in the KEGG metabolic database.

Given a biosynthetic pathway *ρ*(*c*) that produces a compound *c*, we have shown in the previous sections how to estimate the individual contributions to the cost function (Equation 3). By using the cost function, thus, biosynthetic pathways *ρ*(*c*) can be ranked. However, in order to rank all viable biosynthetic pathways *ρ*(*c*) for a compound *c *of interest, the problem of pathway enumeration needs to be addressed. For this purpose, modeling of the metabolic network in the EMRS was done by using directed hypergraphs, where reactions are represented by hyperedges that connect sets of vertices (the substrates) to disjoint sets of vertices (the products) [62]. Directed hypergraph formalism, though more complex than simple-graph models, provides a complete representation of all compounds involved in biochemical transformations. By using the hypergraph formalism, we implemented a retrosynthetic algorithm that enumerates all pathways starting from target compounds of interest. One main advantage of the pathway enumeration in the EMRS is that complexity can be controlled by tuning the atomic height *h *of the molecular signatures. Higher values of *h *imply that the number of pathways between two metabolites is approximately the same as the number of possible pathways in metabolic networks of annotated databases such as KEGG [24] or MetaCyc [25], while lower *h *values generate more novel reactions and therefore more possible pathways are formed between those two metabolites. This result is illustrated for the case of pathway enumeration between chorismate and tyrosine in *E. coli *for different heights *h *using a reaction representation at the level of the 3rd digit in the EC number classification (Figure 4). In general, the possible number of pathways that can be formed between these two metabolites increases exponentially with the number of reaction steps. When values of the molecular signature height are high (*h *≥ 6), new reactions are unlikely to be generated and therefore the number of pathways becomes the same as the number of pathways available in KEGG (the reference metabolic database); whereas as the height *h *decreases, the number of new reactions and, thus, the number of pathways starts growing while getting closer to those results that were obtained in BNICE by using BEM matrices [33].

In a general setting, the problem of finding the optimal pathway ρ*(*c*) in Equation 4 that produces the target compound *c *is equivalent to finding the shortest hyperpath in a weighted hypergraph. This problem is known to be an NP-hard problem [62], although it can be reduced to a polynomially solvable problem if the cost function in Equation 3 is reformulated as an additive objective function [62]. In the EMRS approach, complexity can be controlled by varying the specificity of the molecular signature. This flexibility allows us to follow the strategy of enumerating all biosynthetic pathways *ρ*(*c*) and computing their associated costs *W*(*c*, *ρ*) in Equation 3.

### Validation test for auxotrophic production in *E. coli*

A validation test for the ranking score was carried out by testing its ability to identify native biosynthetic pathways for several essential metabolites (the 20 amino acids, citrate, ATP, ADP, GTP and GDP) in auxotrophic strains of *E. coli*. These strains were rendered unable to synthesize those essential compounds by inactivation of the enzymes that natively produce them. We assumed that native pathways, which have been selectively conserved under evolutionary pressure, are efficient ways to produce the compounds while keeping cell growth. The validation, thus, consisted of checking if the pathways that were ranked at the top of the list correspond to native pathways. In order to find all possible ways to synthesize the amino acids, we ran the retrosynthetic search in these auxotrophic strains. The output results of the search, which are given in Table 2, provided both native and alternative pathways connecting the auxotroph to the compounds. The results of this test showed that in 98% of the cases native pathways were found within the top 10 ranked pathways for each amino acid.

**Table 2:** Native pathway identification in auxotrophic *E. coli*

Compound	Total	Native pathways	% of natives in best 10	*p*-value
Alanine	19	7	60.00%	8.59e-08

Arginine	1	1	100.00%	4.65e-02

Asparagine	3	3	100.00%	8.42e-05

Aspartic acid	7	7	100.00%	6.91e-09

Cysteine	11	5	100.00%	1.32e-07

Glutamic acid	74	53	100.00%	6.41e-52

Glutamine	7	5	100.00%	1.41e-05

Glycine	31	16	80.00%	3.16e-08

Histidine	4	4	100.00%	4.85e-04

Isoleucine	1	1	100.00%	4.55e-02

Leucine	1	1	100.00%	4.54e-02

Lysine	1	1	100.00%	6.38e-02

Methionine	107	106	100.00%	1.08e-259

Phenylalanine	4	1	100.00%	6.38e-02

Proline	1	1	100.00%	2.17e-02

Serine	2	2	100.00%	1.11e-03

Threonine	1	1	100.00%	4.35e-02

Tryptophan	2	2	100.00%	1.89e-03

Tyrosine	2	1	100.00%	1.92e-02

Valine	1	1	100.00%	4.55e-02

Citrate	4	3	100.00%	5.71e-05

ATP	12	6	100.00%	6.73e-06

GTP	2	2	100.00%	2.08-03

ADP	316	204	100.00%	2.89e-169

GDP	283	171	100.00%	*<*1.0e-324

Biosynthetic pathways for the 20 amino acids, citrate and ATP/ADP and GTP/GDP enumerated for *E. coli *strains where enzymes producing the compounds have been deleted. For each test, columns correspond to: total number of enumerated pathways; number of native pathways in the wild-type strain; percentage of native pathways in the top 10 best ranked pathways; and *p*-values for the number of genes in *E. coli *strains top ranked with respect to the total enzyme genes in the database.

An additional validation test was performed in order to evaluate the accuracy of the gene compatibility predictor. The result of this test, summarized in Table 2, showed that genes from the full list in the database that were predicted to be the best candidates to be inserted in the auxotroph strains corresponded significantly (*p*-value *<*0.05) to native genes of *E. coli*. These results are significant since the sequences under test were not part of the training set used for building the gene compatibility predictor. In summary, this test showed that the proposed ranking function can potentially identify heterologous biosynthetic pathways to insert in an organism to produce a desired compound while selecting the ones that are close to native pathways in the chassis.

### The RetroPath webserver

**Figure 5:**
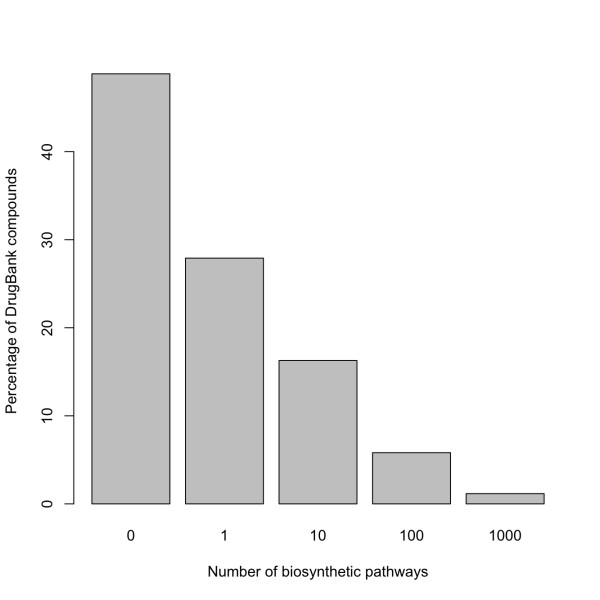
**Retrosynthetic pathways for production of DrugBank compounds in *E. coli***. Percentage of compounds in the DrugBank database with alternative biosynthetic pathways in *E. coli*.

As shown in previous sections, the procedure of pathway selection by retrosynthesis is a complex task that implies the adoption of several design decisions, some of them on a case-by-case basis. In order to help on the decision-making process, we have developed an online tool: the RetroPath webserver that guides the designer through the retrosynthesis process. The design starts by choosing the target compound, which can be given as an SDF molecular file. Additionally, the user decides the level of molecular resolution to use in the molecular signature representation. For instance, we have analyzed the set of molecular structures in DrugBank [63] as initial target compounds. For this set, we found that more than 50% of reactions producing these compounds belong to the *E. coli *EMRS of height *h *= 6. Furthermore, the distribution of the number of alternative biosynthetic pathways in the compound set follows a power law, as it is shown in Figure 5, which means that in some cases there might be thousands of alternative pathways that have to be ranked according to the ranking function in Equation 3 to search for the optimal pathway.

In order to illustrate the design process, we next present three examples of heterologous pathway design using the RetroPath webserver, the production of two *β*-lactams antibiotics in *E. coli *(penicillin G and cephalosporin), and of one antitumor drug (taxol) in yeast.

### Design examples of retrosynthetic pathways

#### Production of β-lactams in E. coli

The industrial production of penicillin G occurs via fermentation using the filamentous fungus *Penicillium chrysogenum*. A recent study has opened up the possibility of producing penicillin G in an organism that is used as a producer of pharmaceuticals; the yeast *Hansenula polymorpha *[64]. Interestingly, the biosynthetic pathways of penicillin G are shared by another *β*-lactam antibiotic, cephalosporin, which is produced in the fungus *Acremonium chrysogenum *and synthetised from isopenicillin N, the penultimate precursor for penicillin production [65].

**Figure 6:**
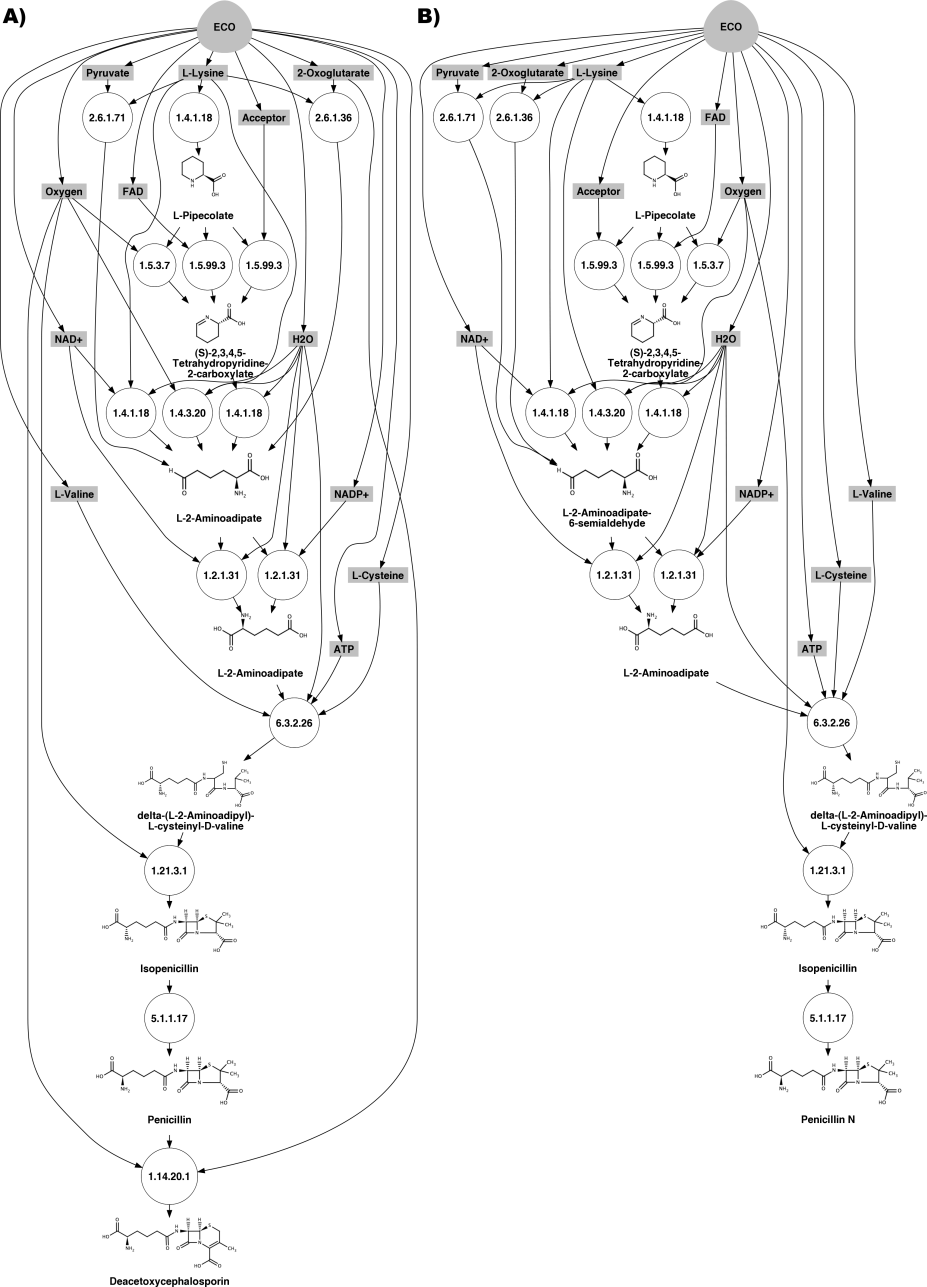
**Retrosynthetic maps for the production in *E. coli *of A) penicillin G and B) cephalosporin**. Compounds in gray are endogenous to the chassis organism (*E. coli*); enzymatic reactions are represented as circles; branching compounds, which can be produced by more than one biochemical transformation, are (S)-2,3,4,5-tetrahydropyridine-2-carboxylate, L-2-aminoadipate-6-semialdehyde; the target compound is at the bottom of the plot.

Using the retrosynthetic method that we have developed, retrosynthetic graphs were generated for *β*-lactam antibiotics, in particular penicillin and cephalosporin (Figures 6A,B). The chosen chassis organism was *E. coli*. Four different pathways were found at a signature reaction height of *h *= 4 for penicillin N production and in particular one involving the nonribosomal peptide synthetase *δ*-(L-*α*-aminoadipyl)-L-cysteinyl-D-valine synthetase (EC 6.3.2.26) and isopenicillin N synthetase (EC 1.21.3.1). These pathways are the same as those that were implemented in the aforementioned studies in yeast and fungi to produce the isopenicillin N. In the cephalosporin biosynthesis pathway the isopenicillin N is converted into penicillin N, itself transformed into deacetoxycephalosporin. The retrosynthetic maps of height *h *= 4 for heterologous production of penicillin N and deacetoxicephalosporin in *E. coli *are shown in Figure 6. In the retrosynthetic graph, the enzyme deacetoxycephalosporin-C synthase (EC 1.14.20.1) is the one responsible of the deacetoxycephalosporin formation. Table 3 ranks the 6 pathways in the map leading to penicillin N according to the cost function in Equation 3. Toxicity values for intermediates were predicted by using our model built from an experimental library of toxicity values in *E. coli *while fluxes were estimated from a reconstructed metabolic model of *E. coli*, as described in the Methods section. The optimal pathway involves five exogenous enzymatic steps, while the alternative pathways involves up to seven steps. In Table 3, the alternative routes for the production of penicillin N are generated by the synthesis step of the precursor L-2-aminoadipate-6-semialdehyde, where the retrosynthetic search identified several enzymatic routes that can be connected to precursors in *E. coli*.

**Table 3:** Ranked pathways for biosynthesis of Penicillin N in *E. coli*

EC number	product	cost (toxicity)	*ρ*_1_	*ρ*_2_	*ρ*_3_	*ρ*_4_	*ρ*_5_	*ρ*_6_
5.1.1.17	C06564	1.17 (2.63)	X	X	X	X	X	X

1.21.3.1	C05557	0.81 (2.94)	X	X	X	X	X	X

6.3.2.26	C05556	2.05 (0.87)	X	X	X	X	X	X

1.2.1.31	C00956	1.09 (0.13)	X	X	X	X	X	X

2.6.1.36	C04076	0.74 (0.72)	X	-	-	-	-	-

1.4.3.20	C04076	1.13 (0.72)	-	-	-	X	-	-

2.6.1.71	C04076	1.34 (0.72)	-	-	X	-	-	-

1.4.1.18	C04076	1.54 (0.72)	-	X	-	-	X	X

1.5.3.7	C00450	4.94 (0.91)	-	-	-	-	-	X

1.5.99.3	C00450	5.30 (0.91)	-	-	-	-	X	-

1.4.1.18*	C00408	1.43 (0.93)	-	-	-	-	X	X

		*v_c_*(*ρ*)	6.56	6.53	6.55	5.55	5.56	5.55

		*W*(*ρ*)	9.33	9.67	9.93	9.74	12.52	16.52

Each row corresponds to the insertion of one enzyme in the pathway in order to produce the given intermediate product in the second column. The estimated cost and product toxicity are given in the third column. The last two rows provide the estimate of maximum flux *v_c_*(*ρ*) for the pathway, and the total cost *W*(*ρ*) according to Equation 3. C06564: Penicillin N; C05557: Isopenicillin; C05556: *δ*-(L-*α*-Aminoadipyl)-L-cysteinyl-D-valine; C00956: L-2-Aminoadipate; C04076: L-2-Aminoadipate-6-semialdehyde; C00450: (S)-2,3,4,5-Tetrahydropyridine-2-carboxylate; C00408: L-Pipecolate. Starred EC numbers correspond to putative enzymes.

#### Production of taxol (paclitaxel) in yeast

**Figure 7:**
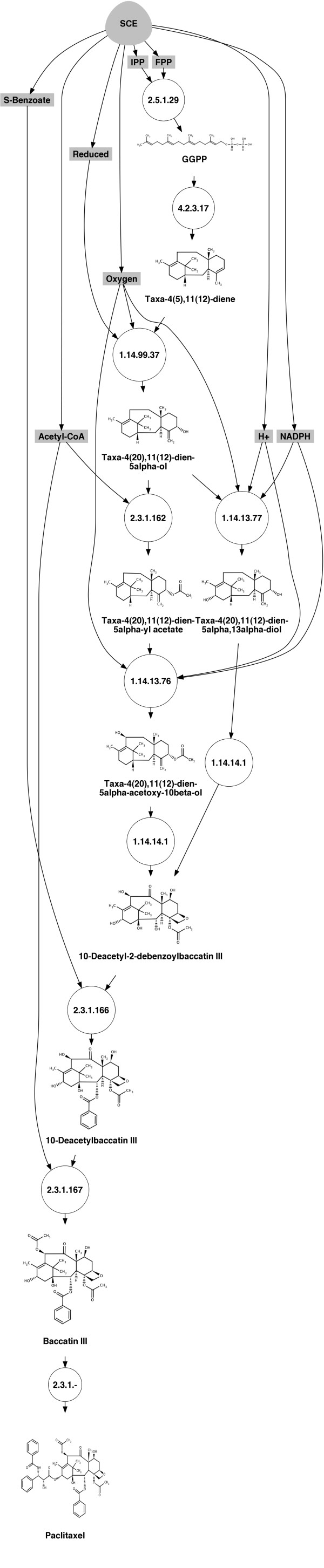
**Retrosynthetic map for the production of paclitaxel (taxol) in yeast**. Compounds in gray are endogenous to the chassis organism (*S. cerevisiae*); enzymatic reactions are represented as circles; 10-deactyl-2-debenzoylbaccatin III appears as a branching compound; the target compound is at the bottom of the plot.

Taxol (paclitaxel) is an anticancer drug first isolated from the Pacific yew tree *Taxus brevifolia*. Today, taxol derives largely by semisynthesis from the advanced taxoid 10-deacetylbaccatin III obtained from the European yew tree *Taxus baccata *[66]. Currently its production has a limiting rate as it depends on plant cell processes as well as chemical and biotechnological semisynthesis processes. For the past few years, a number of studies have been contributing to the elucidation of the biosynthetic mechanism of taxol and efforts have been made in order to attain cost-effective production through heterologous biosynthesis of taxol and its analogues [5]. The retrosynthetic graph for yeast (*Saccharomyces cerevisiae*) which was computed for a signature height *h *= 4, (Figure 7), goes from the isopentenyl to taxol and contains 2 different pathways with 8 and 9 steps, respectively, that share most of the intermediates and only differ at two steps:

1. From isopentenyl (IPP) to taxadien-5*α*-ol: The isopentenyl, native to yeast, undergoes 3 reaction steps to form the taxadien-5*α*-ol. Those are catalyzed by the geranylgeranyl-diphosphate (GGPP) synthase (EC 2.5.1.29) that forms the GGPP (C00353) [67], by the taxadiene synthase (EC 4.2.3.17) that forms taxa-4(5),11(12)diene (C11894) [68] and finally by the taxadiene 5*α *hydrolase (EC 1.14.99.37) that forms the taxadien-5*α*-ol [69]. The first two reactions have been reportedly implemented in *E. coli *[5,70] and, furthermore, were subject to engineering in order to optimize the taxadiene pathway production [5].

2. From the taxadien-5-*α*-ol to taxol: From the taxa-4(20),11(12)-dien-5*α*-ol two pathways are possible, producing either taxa-4(20),11(12)-dien-5*α*,13*α*-diol by the taxane 13*α *hydrolase (EC 1.14.13.77) or the taxa-4(20),11(12)-dien-5*α*-yl acetate by the taxadien-5*α*-O-acetyl transferase [71]. Taxane 10*β *hydroxylase (EC 1.14.13.76) is producing the taxa-4(20),11(12)-dien5*α*,10*β*-diol 5 acetate [72]. This part of the pathway was implemented in the yeast *Saccharomyces cerevisiae *[73]. The next steps described successively the formation of 10-deacetyl-2-debenzoylbaccatin III (C11899), the 10-deacetylbaccatin III (C11700) catalyzed by taxane 2*α*-O-benzoyltransferase (EC 2.3.1.166), and the Baccatin III catalyzed by taxane 2*α*-O-benzoyltransferase [74] to finally form the taxol.

Table 4 ranks the 2 retrosynthetic pathways in yeast leading to taxol production according to the cost function in Equation 3. In this example, toxicity of intermediates was not considered in the ranking (*λ_tox _*= 0) and therefore the optimal weighting terms in Equation 3 were taken accordingly (see details in Methods). The two alternative routes are given by the way the precursor 10-deacetyl-2-debenzoylbaccatin is produced. The optimal pathway involves 8 exogenous enzymes, while the alternative one involves 9 enzymes.

**Table 4:** Ranked pathways for biosynthesis of taxol in *S. cerevisiae*

EC number	product	cost	*ρ*_1_	*ρ*_2_
2.3.1.- *	C07394	1.34	X	X

2.3.1.167	C11900	1.17	X	X

2.3.1.166	C11700	1.07	X	X

1.14.14.1*	C11899	1.78	X	X

1.14.13.76	C11898	5.64	-	X

1.14.13.77	C11897	5.62	X	-

2.3.1.162	C11896	1.06	-	X

1.14.99.37	C11895	5.62	X	X

4.2.3.17	C11894	1.28	X	X

2.5.1.29	C00353	0.69	X	X

		*v_c_*(*ρ*)	0.094	0.093

		*W*(*ρ*)	18.64	19.72

Each row corresponds to the insertion of one enzyme in the pathway in order to produce the given intermediate product in the second column. The estimated cost is given in the third column 3. The last two rows provide the estimate of maximum flux *v_c_*(*ρ*) for the pathway, and the total cost *W*(*ρ*) according to Equation 3. C07394: Paclitaxel; C11900: Baccatin; C11700: 10-Deacetylbaccatin; C11899: 10-Deacetyl-2-debenzoylbaccatin; C11898: Taxa-4(20),11(12)-dien-5*α*-acetoxy-10*β*-ol; C11897: Taxa-4(20),11(12)-dien-5*α*,13*α*-diol; C11896: Taxa-4(20),11(12)-dien-5*α*-yl; C11895: Taxa-4(20),11(12)-dien-5*α*-ol; C11894: Taxa-4(5),11(12)-diene; C00353: Geranylgeranyl. Starred EC numbers correspond to putative enzymes.

## Conclusions

We presented in this work an automated protocol to assist synthetic biologists and metabolic engineers in the design and insertion of efficient heterologous biosynthetic metabolic pathways in chassis organisms. Our method is based on the retrosynthesis algorithm, an idea borrowed from the allied field of synthetic chemistry. In order to perform this analysis in metabolic networks, we used the molecular signature descriptor, a 2D representation of molecular graphs that provides a characterization of compounds and reactions in the network. Molecular signatures provide a homogeneous way to represent through hypergraphs the set of chemical species and transformations present in cell's metabolism. The representation in the molecular signature space is also an efficient way to measure chemical similarity between compounds and reactions. We use this framework to develop an algorithm to generate putative novel reactions between compounds in the network, leading to the extended metabolic reaction space (EMRS). The algorithm consists of searching for all combinations of compounds with the same signature as known metabolic reactions.

In order to explore new biosynthetic pathways for compounds, we implemented a retrosynthetic algorithm in the EMRS to build the map of all reachable compounds from the chassis organism through biochemical transformations. The complexity of the retrosynthetic search, a problem that has been limiting so far the adoption of the retrosynthetic approach into the manufacturing pipeline, has been efficiently addressed in our method through the atomic height *h *of the molecular signature. As *h *increases, the number of possible pathways converges to the annotated pathways in metabolic databases. Lowering *h*, in turn, leads progressively to a combinatorial explosion with multiple alternative pathways containing putative reactions, as the ones obtained in the bond-electron and atom tracking models of the metabolic graph.

The retrosynthetic map contains both annotated and putative reactions catalyzed by identified exogenous enzymes, providing several alternative pathways leading to the target compound. We associated a cost of insertion to each pathway based on several criteria such as gene insertion cost, expression levels, enzyme efficiency and nominal fluxes. Furthermore, an algorithm similar to the shortest pathway search has been implemented in order to rank all possible pathways. We showed that the distribution of alternative biosynthetic pathways for *E. coli *in the list of compounds of medicinal interest in DrugBank follows a power law, being in some cases in the order of thousands. Therefore, it is necessary to implement an efficient ranking function as the one presented here in order to select the best heterologous pathways to insert in the chassis organism. For instance, we applied the retrosynthetic algorithm in order to search for heterologous biosynthetic pathways for two compounds in DrugBank: penicillin N and taxol. In both cases, several alternative pathways for bioproduction were found. The identified pathways contained both known biochemical transformations previously reported as well as other alternative pathways. In order to select the best combinations to engineer, pathways were ranked according to several cost factors such as number of inserted enzymes, gene compatibility, toxicity, and nominal fluxes. The individual contribution of these factors to the ranking function was optimally adjusted so that native pathways were ranked first with respect to predicted pathways. Our multi-criteria approach can be easily tuned depending on the data available for organisms, as it was illustrated by the optimal adjustment of the weighting parameters for different combinations of factors. The ability of the ranking function to identify native pathways was tested and validated in the case of biosynthetic pathways of several essential metabolites (amino acids, citrate, ATP/ADP, GTP/GDP) in auxotrophic strains of *E. coli*. In this test, native enzymes were correctly ranked by means of our methodology at the top of the enumerated biosynthetic pathways.

Even though our methodology searches for enzymes providing the best performance for the overall process, we might be interested in some cases in increasing the efficiency levels for some of the promiscuous reactions involved in the pathway due to their poor performance, a rate-limiting factor in the production of the target compound. In that case, it would be necessary to introduce mutations in order to re-engineer enzyme variants with detectable levels of the desired catalytic activity. Using protein molecular signatures and kernel methods, we already proposed a methodology to search for promiscuity hot-spot residues in the enzyme sequence and outlined a method to find variants with enhanced promiscuity levels [46], which might be applicable in this case. Therefore, this work provides a full biosynthetic automated pipeline for the design and production of therapeutics and other compounds in flexible on-demand cell factories. Going beyond classical metabolic engineering, our synthetic biology approach meets the expected requirements of reusability and modularity that will become integral part of next generation biosynthetic devices.

## Methods

### Definitions

#### Atomic signature

Let *G *= (*V*, *E*) be a molecular graph, where vertices *V *correspond to atoms, and edges *E *to bonds. An atomic signature is a canonical representation of the subgraph of *G *surrounding a particular atom *x *∈ *V *. This subgraph includes all atoms and bonds up to a predefined distance from the given atom, the signature height *h*.

#### Molecular signature

The molecular signature is a vector whose components are represented in the space defined by a basis formed by atomic signatures. Initially developed for chemicals [39], the signature molecular descriptor was later extended to protein sequences [37,75]. Each component of a molecular signature counts the number of occurrences of a particular atomic signature in the molecule. If *G *= (*V*, *E*) is a molecular graph, where vertices *V *correspond to atoms, and edges *E *to bonds, then the molecular signature of *G *is given by:(5)

where *^h^σ*(*x*) is the atomic signature of *G *rooted at atom *x_i _*of height *h*.

#### Reaction signature

We assume that enzymatic reactions take the general form: *r *: *s*_1_*S*_1 _+ ... + *s_n_S_n _*→ *p*_1_*P*_1 _+ ... + *p_m_P_m _*, where *s_i _*and *p_j _*are the stoichiometric coefficients of substrates *S_i _*and products *P_j_*. The signature of reaction *R *of height *h *is defined by the vector:(6)

#### Metabolic reaction space

In a metabolic network, the reaction space *R *is formed by the set of reactions *r *in the network, which are defined as ordered pairs of substrates *s *∈ *C *and products *p *∈ *C *belonging to the metabolite space *C*:(7)

#### Metabolic reaction signature space

The signature reaction space *^h^σ*(*R*) of height *h *is given by mapping of the set of metabolic reactions *r *∈ *R *into signature reactions *^h^σ*(*r*) according to Equation 7. **Extended metabolic reaction space (EMRS)**. The extended metabolic reaction space generated by signatures of height *h*, *^h^σ *^-1^(*R*), corresponds to the inverse mapping from the signature space into the reaction space. Since the projection of *R *into the signature space *^h^σ *(*R*) involves some degeneracy, the regeneration of the metabolic map creates new putative reactions consisting of combinations of substrates and products that verify the reaction signatures in addition to the nominal ones:(8)

#### Reaction chemical similarity

We define a measure of chemical similarity in the signature space between reaction signatures of height *h *by using the Tanimoto similarity coefficient:(9)

where operations are applied into the vector space determined by the net difference of the signatures of products and substrates (Equation 7).

This similarity measure focus on the reaction centers that define the chemical transformation rather than on the full atomic structure. As the height *h *is increased up to the maximum diameter of the graph or canonical signature, the similarity measure extends further up to the rest of the molecular structure. By definition, two reactions *r *and r* that share the same signature up to some height *h *possess identical signatures up to that molecular resolution *h*:(10)

#### Exogenous biosynthetic pathway

An exogenous biosynthetic pathway *ρ *∈ *ρ*(*c*) for a target compound *c *∈ *C *is defined as a collection of reactions {*r*_1_, *r*_2_, ..., *r_n_*} in the EMRS that connects metabolites in the chassis organism to the product *c *through biochemical transformations.

### Ranking terms

**Reaction thermodynamic feasibility **was computed through the estimation of Gibbs energy of the reaction by using a group contribution approach [43,50]. This method considers the Gibbs energy of each metabolite species as the sum of the contributions of their constituents structural subgroups, estimated by linear regression from experimental data. We used the dataset given in [43] in order to compute metabolite Gibbs energy, whereas the reaction Gibbs energy is computed as the energetic balance between its products and substrates:(11)

where *n_i _*is the stoichiometric coefficient of each species, and Δ*G_i _*their estimated Gibbs energy.

**Enzyme promiscuity **was estimated by a support vector machine that was trained from the string or *k*-mer spectra [76] of enzyme sequences *^k^σ*(*S*) in KEGG [24] as inputs by defining the following kernel function:(12)

Enzyme promiscuity in the training set was defined by comparing the chemical similarity of reactions catalyzed by the enzyme sequence *S*, as in [46]:(13)

**Reaction clustering **of the reaction signature space was performed by a hierarchical agglomerative algorithm using as distance metrics the chemical dissimilarity between reactions *d*(*r_i_*, *r_j_*):(14)

The optimal partition of the reaction signature space into *C_i_*, *i *= 1...*n *clusters was determined by the maximum average silhouette [77].

**Enzyme-metabolite interaction **prediction was computed within a given signature reaction space cluster by using a kernel approach known as tensor product [51]. For each reaction cluster, a training set was built consisting of pairs of known enzyme sequences and reactions annotated in KEGG. This dataset was used in order to train a support vector machine defined by the following kernel function:(15)

where *^k^K*(*S_i_*, *S_j_*) is the sequence string kernel defined in Equation 12 and *^h^k*(*r_i_*, *r_j_*) is given by the reaction similarity matrix computed by using the reaction chemical similarity *s*(*r_i_*, *r_j_*) defined in Equation 14. **Enzyme performance **was estimated through a decision tree algorithm implemented for each reaction cluster, as in [53]. Performance data were based on the experimental kinetic constants *k_cat _*and *K_M _*provided by the BRENDA database [54]. Input features consisted of chemical descriptors of substrates and products in reactions and protein sequence descriptors.

#### Gene compatibility

Sequence descriptors were computed from the EMBOSS package of sequence analysis [78]. Phylogenetic distance between the source organism and chassis organism was computed from KEGG taxonomy. These descriptors were computed for the entire KEGG database of non-redundant enzyme sequences and then used to train support vector machine-based predictors for the chassis organisms of interest (*E. coli *and yeast). The training set consisted of a balanced positive set, formed by the list of sequences in the chassis strains, and a negative set formed by sequences selected randomly from the list of organisms other than the chassis. In the model of *E. coli*, we found that the average score for positive hits had a *z*-score = 6.12 (*p*-value = 9.56e-10) for a positive set of 24,894 sequences in E. coli strains among the total set of 681,518 sequences. We used this predictor in order to rank the annotated genes for a given enzyme class, where a *p*-value was associated to each predicted gene by computing the probability of ranking that gene in the given percentile if it were picked at random from the list of genes.

**Compound toxicity **in the chassis organism *E. coli *was estimated through a partial least squares structure-activity relationship model implemented from an in-house database for *E. coli *of 150 compounds with experimentally determined IC50 (half maximal inhibitory concentration). Input descriptors of the model are given by the following molecular descriptors: molecular weight; solubility; average bond length; partition coefficient; molecular surface; and molecular signatures of the compounds.

**Nominal fluxes v **were predicted by using a reconstructed metabolic model of *E. coli *[79] and yeast [80] in the COBRA toolbox [81]. For a given target compound *c *∈ *C *and a putative exogenous biosynthetic pathway *ρ*, an augmented model of the metabolic phenotype of the engineered strain was built from the reference model. The nominal flux of the desired compound *v_c_*(*ρ*) in the augmented model was obtained through linear programming optimization of the stoichiometric mass balance subject to the following constraints:(16)

where **S **is the stoichiometric matrix, *Z *is the objective function of maximizing the biomass formation (growth) rate, *f *(*v_c_*(*ρ*), *Z*) is a definite positive function that monotonically increases with both *v_c_*(*ρ*) and *Z*, and *α*, *β *are the model flux constraints [79]. The chosen objective function in Equation 16 was *f *(*v_c_*(*ρ*), *Z*) = *v_c_*(*ρ*) · *Z*, although in general other objectives might be possible as well (see for instance in [82]).

#### Parameter optimization

Weighting parameters (*λ*_flux_, *λ*_path_, *λ*_tox_) in the cost function given by Equation 3 are adjusted by optimization. The chosen criteria is that pathways that are fully annotated in KEGG should be ranked first with respect to other pathways based solely on predictions. Our approach is similar to the one proposed in [38], although the main difference here is that we optimize the three parameters simultaneously for all metabolites in KEGG by using an estimate of ranking accuracy for each pathway within the full set of enumerated pathways in the EMRS. For a pathway *ρ *∈ *ρ *(*c*) producing a given compound *c*, we define its ranking accuracy as:(17)

where *n_TP _*are the number of pathways of *ρ *(*c*) in KEGG that are ranked at the same or higher score than *ρ*, *n_TN _*are the number of pathways of *ρ*(*c*) not in KEGG which are ranked below *ρ*, and |*ρ*(*c*)| are the total number of pathways producing *c*.

The objective is to maximize the overall aggregate sum of *y_ρ _*extended to the list of enumerated pathways in the EMRS:(18)

For the ranking optimization problem, parameters (*λ*_path_, *λ*_tox_, *λ*_flux_) are not independent since a simultaneous increase of the same magnitude in the three parameters would leave the ranking unchanged. Therefore, in order to solve the problem, we need to fix at least the value of one of the parameters, for instance taking *λ*_path _= 1.0 and *ω_p _*= *ω_e _*= 1. We give three possible solutions depending whether both toxicity and fluxes estimates are available or only one of them (see parameter variations in Additional file 1 Figures S1, S2 and S3): without considering fluxes (*λ*_flux _= 0.0), the optimal value for the toxicity parameter was ; without considering toxicity , the optimal value for the flux parameter was ; finally considering both toxicity and fluxes, the optimal values were obtained at , .

## Competing interests

The authors declare that they have no competing interests.

## Authors' contributions

JLF designed the retrosynthesis overall process. PC designed the experiments and pathway ranking strategy, collected the results, and wrote parts of the software. AGP designed and carried out the toxicity experiments, participated in the design of the pathway ranking strategy, and analyzed the retrosynthetic examples. DF developed the algorithms. PC, AGP, DF, and JLF wrote the manuscript. All authors read and approved the final manuscript.

## Supplementary Material

Additional file 1**Pathway ranking accuracies for different values of parameters (*λ***_**tox**_**, *λ***_**flux**_**)**. Figure S1 plots pathway ranking accuracy for different values of parameter *λ*_tox _without considering fluxes (*λ*_flux _= 0); optimal value is obtained for . Figure S2 plots pathway ranking accuracy for different values of parameter *λ*_flux _without considering toxicity (*λ*_tox _= 0); optimal value is obtained for . Figure S3 plots pathway ranking accuracy for different values of parameters (*λ*_tox_, *λ*_flux_); optimal values are (, ).Click here for file
