# Supplementary material for: A retrosynthetic biology approach to metabolic pathway design for therapeutic production
Source: BMC Syst Biol. 2011 Aug 5;5:122. doi: 10.1186/1752-0509-5-122 (PMC3163555; doi:10.1186/1752-0509-5-122)
Supplement: Additional file 1 — Pathway ranking accuracies for different values of parameters (λtox, λflux). Figure S1 plots pathway ranking accuracy for different values of parameter λtox without considering fluxes (λflux = 0); optimal value is obtained for . Figure S2 plots pathway ranking accuracy for different values of parameter λflux without considering toxicity (λtox = 0); optimal value is obtained for . Figure S3 plots pathway ranking accuracy for different values of parameters (λtox, λflux); optimal values are (, ). [file 1752-0509-5-122-S1.PDF]

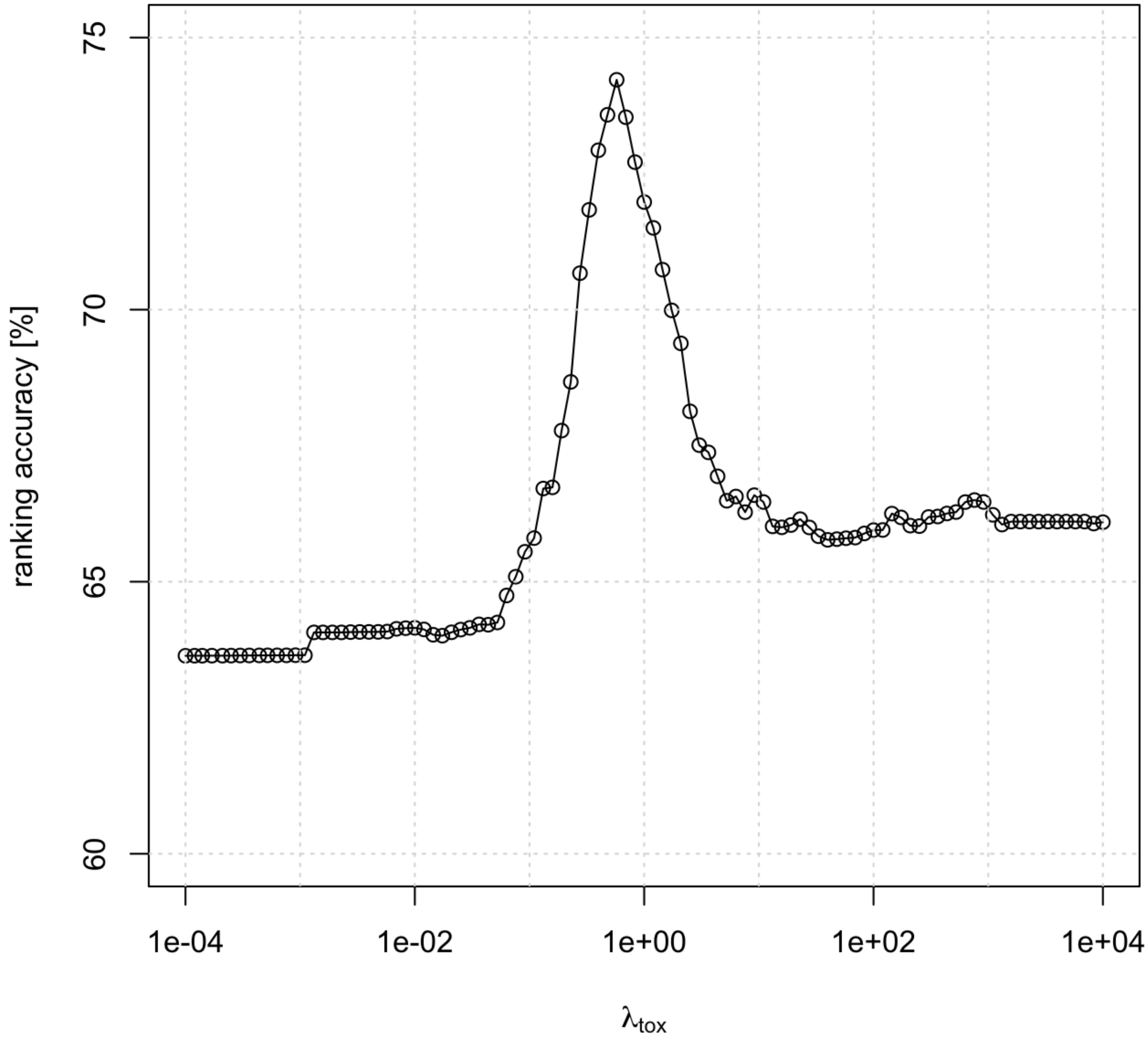


Figure S1. Pathway ranking accuracy for different values of parameter $\lambda_{\texttt{tox}}$ without considering fluxes ($\lambda_{\texttt{flux}} = 0$). Optimal value is obtained for $\lambda^*_{\texttt{tox}} = 0.575$.

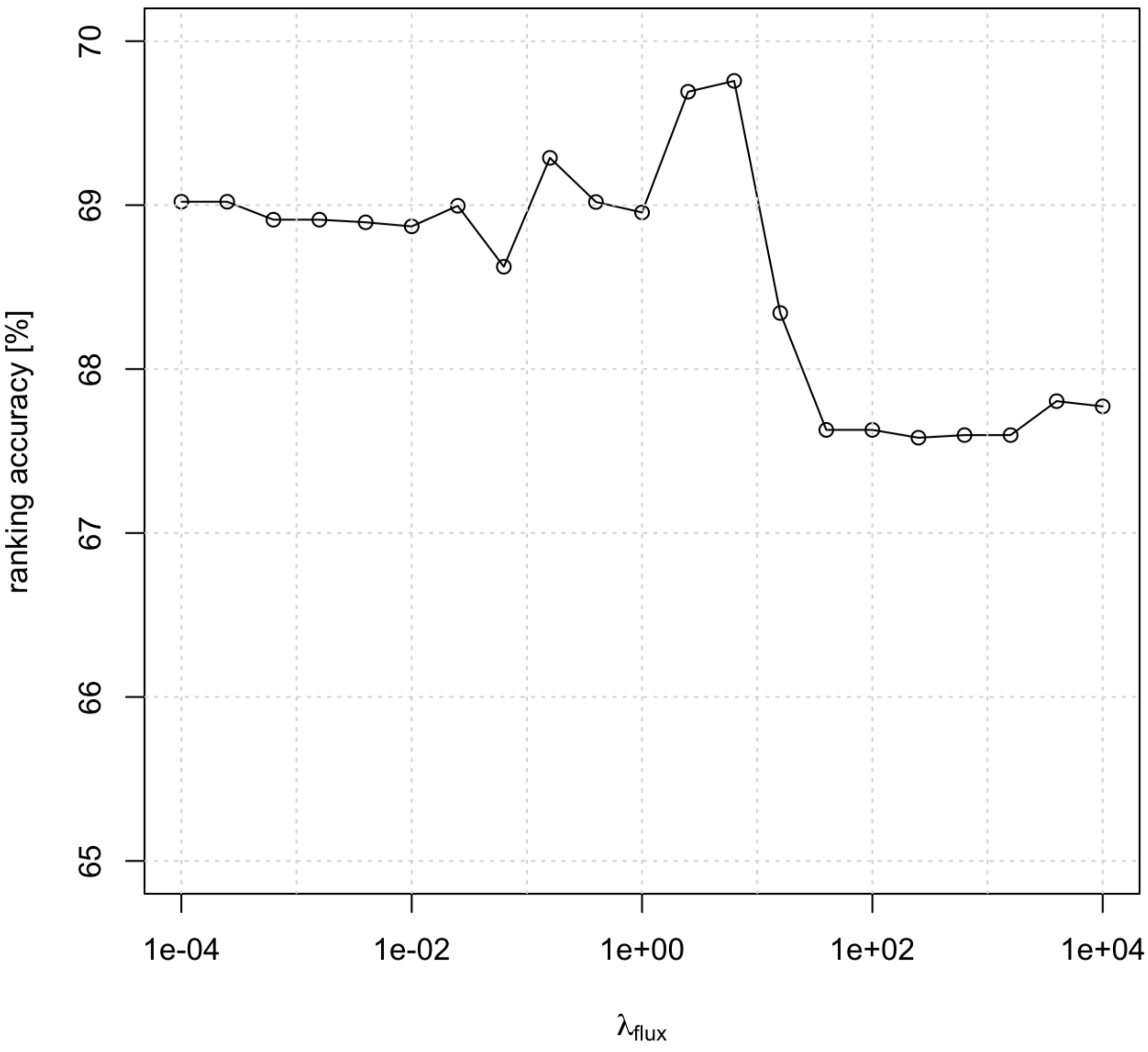


Figure S2. Pathway ranking accuracy for different values of parameter $\lambda_{\texttt{flux}}$ without considering toxicity ($\lambda_{\texttt{tox}} = 0$). Optimal value is obtained for $\lambda^*_{\texttt{flux}} = 0.800$.

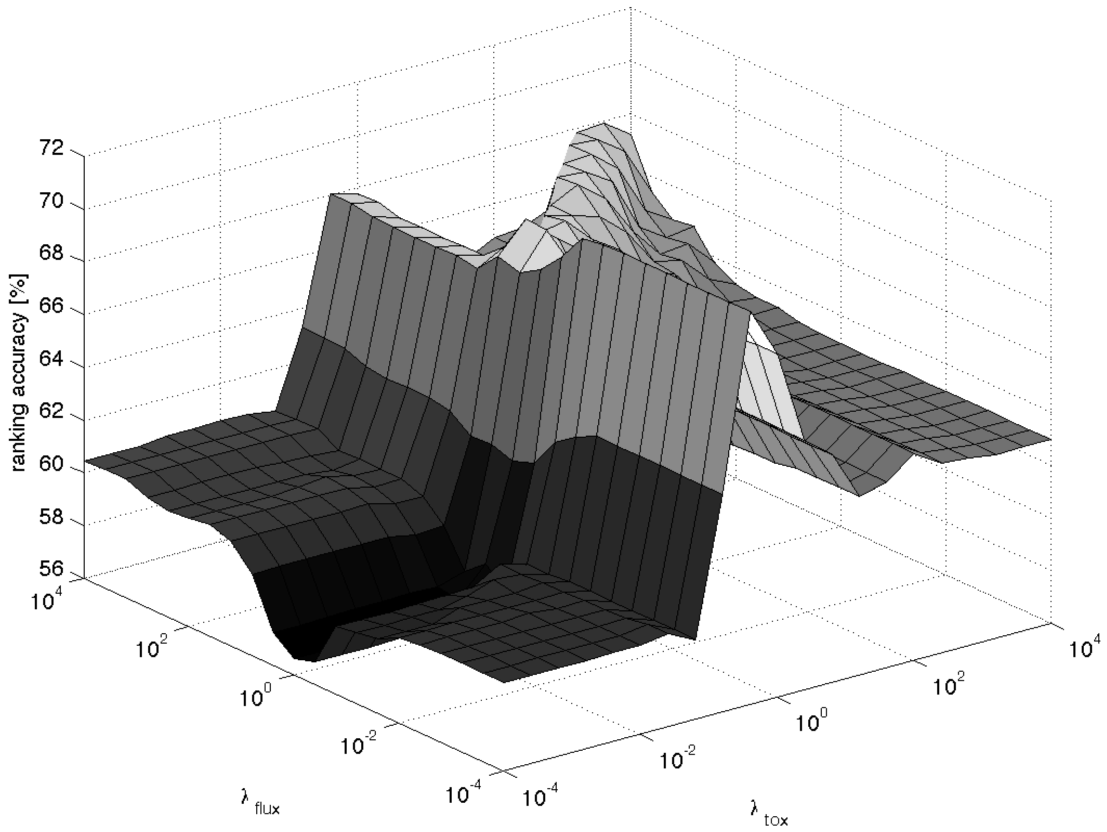


Figure S3. Pathway ranking accuracy for different values of parameters ($\lambda_{\mathtt{tox}}, \lambda_{\mathtt{flux}}$). Optimal values are ($\lambda^*_{\mathtt{flux}} = 0.025, \lambda^*_{\mathtt{tox}} = 0.398$).
